# Whole Exome Sequencing in Vaccine-Induced Thrombotic Thrombocytopenia (VITT)

**DOI:** 10.1155/2024/2860547

**Published:** 2024-07-14

**Authors:** Betti Giusti, Elena Sticchi, Tommaso Capezzuoli, Rebecca Orsi, Lapo Squillantini, Marco Giannini, Samuele Suraci, Angela Antonietta Rogolino, Francesca Cesari, Martina Berteotti, Anna Maria Gori, Elena Lotti, Rossella Marcucci

**Affiliations:** ^1^ Department of Experimental and Clinical Medicine University of Florence, Florence, Italy; ^2^ Atherothrombotic Diseases Center Careggi University Hospital, Florence, Italy

**Keywords:** blood coagulation, COVID-19 vaccines, platelet aggregation, thrombocytopenia, whole exome sequencing

## Abstract

**Background:** In February 2021, a few cases of unusual, severe thrombotic events associated with thrombocytopenia reported after vaccination with ChAdOx1 nCoV-19 (Vaxzevria) or with Johnson & Johnson's Janssen vaccine raise concern about safety. The vaccine-induced thrombotic thrombocytopenia (VITT) has been related to the presence of platelet-activating antibodies directed against platelet Factor 4.

**Objectives:** We investigated VITT subject genetic background by a high-throughput whole exome sequencing (WES) approach in order to investigate VITT genetic predisposition.

**Methods:** Six consecutive patients (females of Caucasian origin with a mean age of 64 years) were referred to the Atherothrombotic Diseases Center (Department of Experimental and Clinical Medicine, Azienda Ospedaliero-Universitaria Careggi, Florence) with a diagnosis of definite VITT underwent WES analysis. WES analysis was performed on the Illumina NextSeq500 platform.

**Results:**WES analysis revealed a total of 140,563 genetic variants. Due to VITT's rare occurrence, we focused attention on rare variants. The global analysis of all high-quality rare variants did not reveal a significant enrichment of mutated genes in biological/functional pathways common to patients analyzed. Afterwards, we focused on rare variants in genes associated with blood coagulation and fibrinolysis, platelet activation and aggregation, integrin-mediated signaling pathway, and inflammation with particular attention to those involved in vascular damage, as well as autoimmune thrombocytopenia. According to ACMG criteria, 47/194 (24.2%) rare variants were classified as uncertain significance variants (VUS), whereas the remaining were likely benign/benign.

**Conclusion:** WES analysis identifies rare variants possibly favoring the prothrombotic state triggered by the exposure to the vaccine. Functional studies and/or extensions to a larger number of patients might allow a more comprehensive definition of these molecular pathways.

## 1. Introduction

The wide supply of vaccines against COVID-19 has allowed to drastically change the course of the pandemic, as of December 13, 2022, 63.8% of the world population is fully vaccinated. In February 2021, a few cases of unusual, severe thrombotic events associated with thrombocytopenia reported after vaccination with ChAdOx1 nCoV-19 (Vaxzevria) or with Johnson & Johnson's Janssen vaccine raises concern about safety. On April 7, 2021, the Pharmacovigilance Risk Assessment Committee (PRAC) of the European Medicines Agency (EMA) concluded for the presence of a causal relationship between vaccination with Vaxzevria and the adverse events of thrombosis combined with thrombocytopenia (VITT) [1].

Moreover, data from a systematic review supported the relationship between VITT and adverse outcome occurrence, also proposing a score for predicting VITT mortality (FAPIC score), including fibrinogen levels, age, platelet count, and the presence of intracerebral hemorrhage (ICH) and cerebral venous thrombosis (CVT) [2].

The binomial constituted by thrombocytopenia and thrombosis is very rare in medicine, and quickly HIT emerged as the most plausible among the clinical pictures characterized by these two signs. Indeed, also in VITT, we identified anti-PF4 antibodies as key elements in the pathophysiological mechanism of the disease [3, 4].

Data from literature published in 2021 defined VITT in terms of five criteria: onset of symptoms 5–30 days after vaccination against SARS-CoV-2, presence of thrombosis, presence of thrombocytopenia (platelet count < 150,000 per cubic millimeter), D-dimer level exceeding 4000 equivalent fibrinogen units (FEU), and presence of antibodies against PF4 detected by enzymatic immunosorbent assay (ELISA) [5]. This condition was mainly observed in women under 55 years of age between 4 and 16 days after administration of the Vaxzevria vaccine or, with an even higher incidence, the Janssen vaccine (Ad26COV2) [1, 6]. According to the EMA, the incidence of VITT in people over 60 years of age is about 1.02 cases per 100,000 doses of vaccine, and this incidence increases by about 1 in 50,000/75,000 doses in people under 60 years of age [7].

In VITT, after vaccine administration, platelet Factor 4 (PF4) likely binds to certain components of the vaccine or the adenoviral vector itself, forming complexes that become neoantigens and induce the formation of antibodies against itself. Similarly to HIT, vaccine-PF4-IgG immune complexes activate platelets through their Fc*γ*RIIA receptors, causing their activation, aggregation, and further release of PF4, leading to a positive feedback cycle of further platelet activation. In addition, these complexes also activate monocytes, which release tissue factor and promote the associated activation of coagulation. Here, too, the once-triggered reaction feeds on itself [8]. Alternatively, a possible trigger for the production of antibodies directed against PF4 might also be represented by the free DNA present in the vaccine; really, previous data from the literature reported that DNA and RNA, as well as DNA/RNA-based aptamers, should be able to induce PF4 conformational changes that might contribute to expose HIT antigens and, in turn, to induce the formation of antibodies against PF4/heparin complexes in mice [4, 9]. Unlike mRNA vaccines, which directly release mRNA into the cytosol of host cells, where they are translated into spike proteins without splicing reactions, adenoviral vector-based vaccine activity requires the nuclear transcription process, which might generate shorter “spike” protein variants, possibly responsible for soluble forms release able to induce a strong inflammatory process which might promote VITT development. Moreover, the presence of polysorbate as an adjuvant in ChAdOx1 and Ad26.CoV2.S vaccines, representing a nonanionic surfactant able to cross the blood–brain barrier and home-to-brain endothelial cells, if complexed with nanoparticles [10, 11], might also be involved in promoting vaccine-induced complications.

Further molecular mechanisms might also contribute to trigger VITT mechanisms; really, it has been proposed that genetically determined Fc*γ*RIIa increased expression as well as altered glycosylation state of the IgG produced in response to vaccine administration, possibly responsible for a higher reactivity to platelet Fc*γ*RIIa, might also be involved in disease mechanisms [12, 13]. Moreover, it has been also suggested a possible contribution of adenoviral vector outflow into the circulation and/or previous presence of antibodies, cross-reactive to other coronaviruses, determining platelet-activating immunocomplexes formation [14].

To the best of our knowledge, little information is available concerning the relationship between host genetic variability and VITT development. Previous data from the literature suggested the possible contribution of specific HLA alleles or haplotypes in the modulation of anti-PF4/P hyperresponsiveness [15]. Moreover, only a recent case report reported the genetic investigation of a VITT subject by Human Genotyping SARS-CoV-2 Research Array, investigating more than 870,000 single-nucleotide polymorphisms (SNPs), thus hypothesizing the possible contribution of genetic variants in genes involved in coagulation, thrombophilia, inflammation [16].

In the present study, we investigated the genetic background of VITT subjects by a high-throughput whole exome sequencing (WES) approach, in order to investigate the possible contribution of genetic risk factors influencing the development of adenoviral vectors-vaccines adverse events. In particular, in this report, we focused attention on genes selected for their involvement in blood coagulation and fibrinolysis, platelet activation and aggregation, integrin-mediated signaling pathway, inflammation with particular attention to those involved in vascular damage, and autoimmune thrombocytopenia.

## 2. Materials and Methods

### 2.1. Study Population

Six patients referred to the Atherothrombotic Diseases Center (Department of Experimental and Clinical Medicine, Azienda Ospedaliero-Universitaria Careggi, Florence) with a diagnosis of definite VITT underwent molecular characterization through WES analysis. There are six female patients of Caucasian origin with a mean age of 64 years. Of these six patients, five (VITT02, VITT05, VITT06, VITT18, and VITT21) received the Vaxzevria vaccine (AstraZeneca), and one (VITT03) received the JANSSEN vaccine (Johnson & Johnson).

The study was conducted in accordance with the Declaration of Helsinki and was approved by the National Ethical Committee for studies on COVID-19 (approval n. 333 – 2020/2021 Istituto Nazionale per le Malattie Infettive “L. Spallanzani”, Rome) and by the participating center's ethics committees (17104_oss). All cases of VITT were adjudicated independently by three blinded experts (VDS, PG, RM), according to the criteria proposed by Pavord et al. [5].

Table 1 reported clinical data of the six patients with VITT analyzed: arterial and/or venous thrombotic events, hemorrhagic complications, neurological damage, and prognosis.

### 2.2. WES Analysis

Peripheral blood DNA has been isolated using FlexiGene kit (QIAGEN®, Hilden, Germany), according to manufacturer's instructions, and quantified by NanoDrop™ One/OneC Microvolume UV-Vis Spectrophotometer and Invitrogen Qubit 4 Fluorometer (ThermoFisher Scientific, Waltham, MA, USA). The WES analysis has been performed through Illumina Technology. Genomic libraries have been prepared according to SureSelect XT HS protocol (Agilent Technologies, Santa Clara, CA, USA). The pooled libraries were paired-end sequenced on the Illumina NextSeq500 platform (Illumina Inc, San Diego, CA, USA).

Concerning the Assembly and Variant Calling phases, reads were aligned with the human reference hg19 genome using the Burrows–Wheeler aligner [17]. Fast and accurate short read alignment with Burrows–Wheeler transform^16^ was performed, and aligned reads were inspected with the IGV software (Broad Institute) [18]. The variant calling for the identification of nucleotide variants was performed using the Genome Analysis Toolkit HaplotypeCaller Module [19]. Coverage statistics and the average depth of coverage were computed using custom scripts in the R software environment [20].

Rare SNVs and InDels were selected by removing all variants with no functional impact (intronic and missense) and with allelic frequency smaller than 0.0001. For each patient, all genes affected by rare variants were analyzed for overrepresentation analysis by using the “gene list” analysis tool of the Reactome database (reactome.org). Reactome pathways with entities FDR smaller than 0.05 were considered statistically significant.

Genes for variants analysis have been selected according to gene ontology biological processes (blood coagulation [GO:0007596] and fibrinolysis [GO:0042730], platelet activation [GO:0030168] and aggregation [GO:0070527], integrin-mediated signaling pathway [GO:0007229], inflammatory response [GO:0006954], and Human Phenotype Ontology [autoimmune thrombocytopenia: HP:0001973]), as well as according to literature data (Table S1).

Variant prioritization phase was carried out as follows: nonsynonymous variants with a minor allele frequency (MAF) < 0.01 in publicly available resources (dbSNP; in 1000 Genomes Project or Genome Aggregation Database) were considered. The pathogenic effect of the missense/splicing mutations was estimated using PROVEAN, PolyPhen-2, MutationTaster, FATHMM, NetGene2, ASSP, and SpliceAI tools. Attribution of pathogenicity was performed according to the American College of Medical Genetics (ACMG) guidelines [21].

#### 2.2.1. Variant Selection Strategies

Given the large amount of sequence data, we looked for options to streamline variant selection. Using the gnomAD exomes and the gnomAD genome database, we selected only variants with a MAF < 0.01, and specifically, we focused on rare variants identified in genes involved in blood coagulation and fibrinolysis, platelet activation and aggregation, integrin-mediated signaling pathway, inflammation with particular attention to those involved in vascular damage, and autoimmune thrombocytopenia.

## 3. Results

In Table 1, the demographic and clinical characteristics of the 6 sequenced patients are reported. They were all females with a mean age of 64.2 ± 13.8 years. VITT05 (75 yrs) and VITT18 (42 yrs) patients exhibited the worse clinical outcome. VITT02 and VITT06 showed both venous and arterial thrombosis. Only 1, out of 6 patients, showed only arterial thrombosis (VITT18). The other 3 patients showed only venous thrombosis. No demographic and clinical differences were observed between the only subject (VITT03) receiving the JANSSEN vaccine and the other five subjects receiving the VAXZEVRIA vaccine.

**Figure 1:**
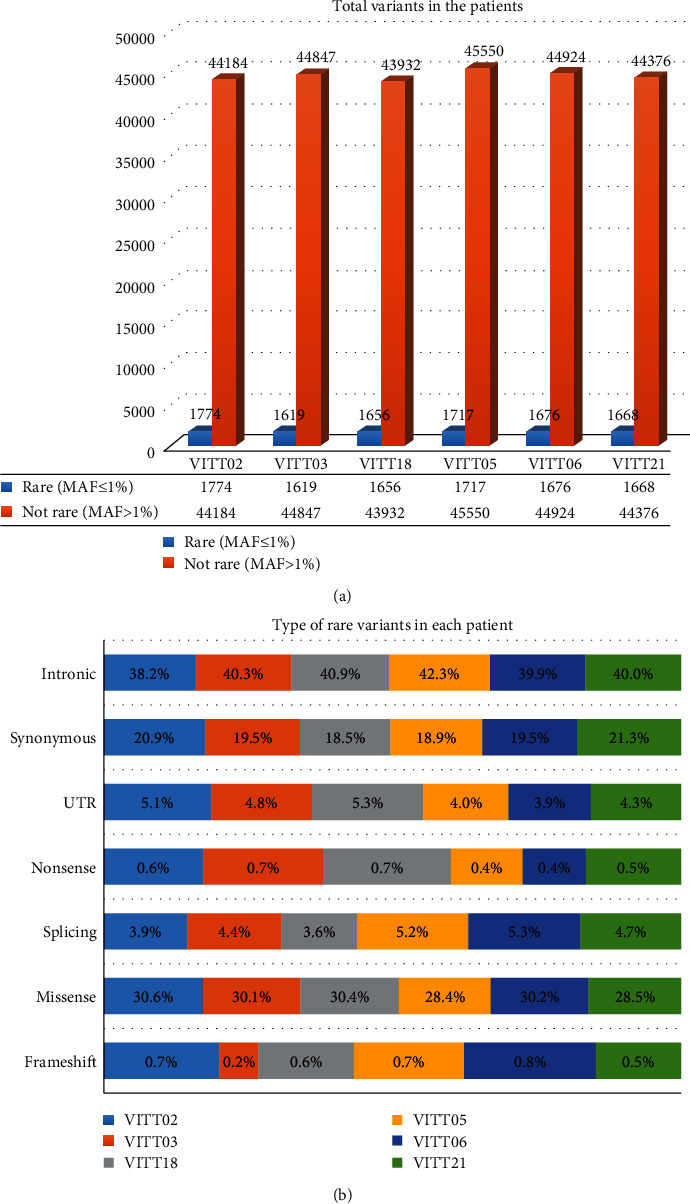
(a) Total variants (rare and not rare) identified in the six patients with VITT; (b) rare variant type (intronic-missense-synonymous-UTR-splicing-frameshift-nonsense) distribution among patients with VITT.

Sequencing analysis of the 6 patients with VITT resulted in a total number of genetic variants of 140,563. The total number of variants per patient analyzed, split into rare (MAF ≤ 0.01) and nonrare variants, is shown in Figure 1(a). The number (Figure 1(a)) and the variant type distribution (Figure 1(b)) are similar among each patient analyzed. Among rare variants, ranging from 1619 to 1774 among the six patients with VITT, the higher percentage is represented by intronic (ranging from 38.2% to 42.3%), missense (ranging from 28.4% to 30.6%), and synonymous variants (ranging from 18.5% to 21.3%), whereas splice sites (ranging from 3.6% to 5.3%), 5′/3′-UTR (ranging from 3.9% to 5.1%), nonsense (ranging from 0.4% to 0.7%), and frameshift (ranging from 0.2% to 0.8%) variants are less frequent (Figure 1(b)).

Due to the rare occurrence of VITT (1/26,000-127,300), we first of all focused the attention on very rare variants in the 6 patients. The global analysis of all high-quality rare variants evaluated by overrepresentation analysis by using the “gene list” analysis tool of the Reactome database did not show a significant enrichment of mutated genes in biological/functional pathways common to the 6 different patients.

Next, we focused attention on rare variants identified in genes selected to be involved in biological/functional processes associated with blood coagulation and fibrinolysis, platelet activation and aggregation, integrin-mediated signaling pathway, inflammation with particular attention to those involved in vascular damage, and autoimmune thrombocytopenia (Table S1).

In Table 2 the patient code, the gene with the reference transcript, the description of the identified variant, the in silico predictors used for the prediction of pathogenicity of missense variants and splicing variants, and variant classification according to ACMG guidelines [21] are reported. Altogether, in the six patients, *n* = 194, rare variants were identified, all at the heterozygous state (Table S2). As ACMG variant classification criteria were considered, *n* = 47, variants (24.2%) have been classified as uncertain significance variants (VUS): 4 in VITT05, 4 in VITT21, 6 in VITT03, 9 in VITT18, 11 in VITT06, and 13 in VITT02 (Table 2). No variant has been classified as likely pathogenic/pathogenic variant. Even if different genetic variants, patient VITT02 and VITT06 carried one of their mutation in the *FGA* gene and VITT02 and VITT18 in the *SERPINA12* gene.

## 4. Discussion

This paper addresses for the first time whether rare genetic variants could contribute to the development of VITT, a clinical condition mainly associated with COVID-19 adenoviral vector vaccine administration and its adverse complications through a WES approach.

Due to the rare occurrence of VITT, in this paper, we focused the attention on rare variants. At a whole analysis of the rare variants identified in the 6 patients with VITT, no significant enrichment of mutated genes in biological/functional pathways common to different analyzed subjects was found. Therefore, we focused on potentially damaging mutations on the basis of ACMG criteria, occurring in selected genes involved in biological and functional processes associated with blood coagulation and fibrinolysis, platelet activation and aggregation, integrin-mediated signaling pathway, inflammation with particular attention to those involved in vascular damage, and autoimmune thrombocytopenia. Actually, selected pathways have been described to contribute to a major push in VITT and in its complication severity [1–4].

For each patient, we identified different VUS ranging from 4 to 13 variants. VITT05 and VITT18 patients exhibited the worst clinical outcome.

In VITT05, a rare VUS in the *STAB2* gene [c.2065C>G (p.Pro689Ala)] has been classified as harmful by most in silico predictors used. *STAB2* gene encodes stabilin-2, expressed predominantly in the sinusoidal endothelium of the liver and spleen and acting as a systemic scavenger receptor for heparin, chondroitin sulfate, dermatan sulfate, nonglycosaminoglycan, acetylated low-density lipoprotein, procollagen propeptides, and advanced glycation end products [22, 23]. Previous WES data also showed an association of *STAB2* locus with venous thromboembolic disease [24]; really, previous data demonstrated that stabilin-2, beyond its role as a scavenger receptor for the aforementioned components, acts as a clearance receptor for VWF, supporting the connection between stabilin-2 and VWF plasma levels [24]. Therefore, it could be hypothesized that *STAB2* genetic variants might affect stabilin-2 cell surface expression by possibly inducing protein misfolding or nonsense-mediated decay, thus influencing molecular pathways stabilin-2 mediated and, in turn, contributing to the development of a prothrombotic phenotype. Indeed, it has been observed a role for stabilin-2 in influencing the thrombus incidence in association with elevated VWF plasma levels, potentially by promoting the interaction of platelets and leukocytes with the vessel wall [25].

VITT05 patient was also a carrier of another rare variant in the *CD47* gene [c.311A>G (p.Asp104Gly)], encoding the leukocyte surface antigen CD47, for which a variable prediction of pathogenicity by in silico tools was evidenced. The contribution of the *CD47* gene might be considered critical, as well; really, the leukocyte surface antigen CD47 plays a role as a receptor for thrombospondin, a secreted matricellular protein observed to be upregulated in vascular cells after injury as well as in chronic disease, and modulator of integrin signaling. Indeed, the CD47-TSP1 binding has been previously shown to influence thrombus formation leading events, including the adhesion of platelets to the endothelial wall, nitric oxide/cGMP (cyclic guanosine monophosphate) signaling induction, platelet activation, and aggregation [26–28]. VITT05 also exhibited two further VUS in genes involved in inflammation pathways. A rare variant in *COL6A1* gene [c.2191C>T (p.Arg731Cys)], encoding for alpha-1 chain of collagen VI, for which in silico tools used for pathogenicity prediction established a damaging effect, was detected. Data from the literature showed that the *COL6A1* gene was upregulated in patients with nonemphysematous chronic obstructive pulmonary disease, so supporting the profibrotic mast cell phenotype, through collagen VI deposition [29]. Moreover, a further variant was found in the *IL5RA* gene [c.913G > A p.(Asp305Asn)], encoding the interleukin-5 receptor subunit alpha, involved in the survival, differentiation, and chemotaxis of eosinophils, contributing to modulate the innate immune system [30].

VITT18 is a patient with a severe disability as a consequence of cerebral stroke with intraparenchymal hemorrhage. Interestingly, this patient carries a missense variant [c. 830G>A (p.Arg277Gln)] in *GP6* gene encoding for platelet glycoprotein VI (GPVI), a collagen receptor involved in collagen-induced platelet adhesion and activation, contributing to platelet procoagulant activity and thrombin and fibrin formation. The GPVI represents a receptor expressed on platelets and megakaryocytes and acts as a receptor for collagen as well as several plasma and vascular proteins (i.e., laminin, fibronectin, and galectin-3). Data from the literature suggested the contribution of platelet GPVI in influencing venous thrombotic complications; really GPVI might sustain the thrombo-inflammatory status by inducing neutrophil granular release. Moreover, it has been suggested the role of a GPVI-thrombin-fibrin feedforward loop supporting the thrombus formation [31]. Apart from the *GP6* gene variant, VITT18 patient also carries two rare missense variants in *SERPINA12* and *MFSD2B* genes [c.1062C>G (p.His354Gln) and c.1000C>G (p.Pro334Ala)], both classified as damaging by all in silico prediction tools. *SERPINA12* gene product, vaspin, is a member of the large family of serine protease inhibitors and has been observed to inhibit target protease kallikrein 7 (KLK7), the process accelerated by the presence of heparin [32]. Actually, it has been evidenced that serum vaspin is an independent prognostic marker of major adverse cardiac events (cardiovascular death, recurrent acute myocardial infarction (AMI), or hospitalization for heart failure) in AMI patients. In particular, low vaspin levels have been found to be associated with increased inflammation in AMI patients [33].

As concerns *MFSD2B*, it encodes the sphingosine-1-phosphate transporter, able to mediate the export of sphingosine-1-phosphate in red blood cells and platelets [34]. A mouse model highlighted that *Mfsd2b* knock-out could influence platelet intrinsic functions, thus supporting its possible role in limiting thrombus formation [35]. In particular, the mouse model suggested that sphingosine-1-phosphate signaling could be involved in platelet biogenesis. Mfsd2b has been observed to be required for sphingosine-1-phosphate release in resting and activated platelets, really *Mfsd2b* knock-out is associated with a reduced platelet intrinsic function and, in turn, a reduced thrombosis in mice. Therefore, gain-of-function variants in the *MFSD2B* gene might contribute to a prothrombotic phenotype. VITT18 phenotype might be also supported by the presence of two further VUS: a variant in the *BCL6* gene, encoding a transcriptional repressor, able to influence T cell proliferative capacity and fate, and a variant in *TRPV1* gene, encoding a ligand-activated nonselective calcium permeant cation channel involved in the detection of noxious chemical and thermal stimuli. Data from mouse models reported that Bcl6 expression was reduced in the presence of mir-155 (microRNA 155) deficiency in advanced atherosclerosis, supporting its involvement in the modulation of the atherosclerotic process [36]. Moreover, BCL6 has been observed to be involved in live-attenuated influenza vaccine (LAIV)-induced T follicular helper cell differentiation, influencing the antibody response [37]. As concerns the *TRPV1* gene, it has been shown that brain channels are activated by ischemic stroke, causing neurological and motor deficits as well as infarction after brain ischemia [38]. Moreover, it has been reported that TRPV1 is present in platelets, thus representing a substrate for platelet activation by inflammatory mediators in atherosclerotic plaques [39].

In VITT02 patient, in whom both venous and arterial thrombosis occurred, a VUS with all damaging predictors of pathogenicity [c.608T>C (p.Leu203Pro)] was found in the *SERPINA12* gene. In addition, in VITT02 patient, there has been evidence of the presence of VUS in genes involved in two strictly related biological processes: (cell-matrix adhesion [*ITGA2B*, *ITGAD*, and *FGA*, gene ontology term GO:0007160] and blood coagulation [*THBD* and *FGA*, GO:0072377]). Interestingly, VITT02 exhibited the presence of two variants in genes encoding for integrin family members, such as *ITGA2B*, encoding for the integrin alpha-IIb and associated with Glanzmann thrombasthenia (OMIM 273800), and *ITGAD*, encoding for the integrin alpha-D. Altogether, these loci might contribute to explain the thrombotic profile. Among other variants identified, two have not been previously described in the general population; the first one is a rare missense variant (c.1465G>C) in *THBD* gene, encoding for thrombomodulin, an endothelial-specific type I membrane receptor involved in the regulation of the coagulation process, determining the substitution of the Aspartic Acid at position 489 with an Histidine (p.Asp489His) located in the extracellular region in the serine/threonine-rich domain, a binding side for alpha-L/beta-2 and alpha-M/beta-2 integrin [40]; the second one is a rare missense variant [c.1132T>A (p.Ser378Thr)] in the *FGA* gene, encoding for the fibrinogen *α*-chain. As concerns the *THBD* variant, the serine/threonine-rich domain represents a critical domain for protein anticoagulant activity, and data concerning other variants previously identified in the same domain possibly support its role. Further VUSs have been detected in the *PIEZO1* gene, encoding the piezo-type mechanosensitive ion channel component 1, which was shown to be a key modulator of abnormal platelet activation and thrombosis under hypertension [41], and *GLCE* gene, encoding the D-glucuronyl C5-epimerase, involved in heparan sulfate/heparin biosynthesis [42]. In VITT02 patient, uncertain significant rare variants have also been detected in the *CARD8* and *EGFR* genes, both involved in inflammation pathways. CARD8 protein represents an inflammasome sensor, mediating the activation of the inflammasome according to pathogen-associated signals response. In particular, it has been reported that CARD8 participates in the regulation of cytokines and chemokines expression in endothelial cells and atherosclerotic lesions [43]. As concerns the *EGFR* gene, it encodes a receptor tyrosine kinase binding ligands of the EGF family. Data from RNA sequencing analysis, performed in glioblastoma tissue specimens according to the thrombotic phenotype, showed a higher *EGFR* expression in the group with many thrombi with respect to that with few thrombi [44].

Similarly to VITT02, a VUS in the *FGA* gene was also detected in VITT06 patient, also exhibiting both venous thrombosis and stroke. The missense variant identified in this patient [c.2068C>G (p.Gln690Glu)] has not previously been described and exhibited a variable pathogenicity prediction with the in silico tools used, thus supporting the possible contribution of fibrinogen chain alterations to a prothrombotic phenotype. This patient also exhibited VUS in *VASP*, *TBXAS1*, and *ENTPD1* genes. *VASP* gene encodes the vasodilator-stimulated phosphoprotein, actin-associated proteins involved in a range of processes dependent on cytoskeleton remodeling and cell polarity; in particular, it regulates actin dynamics in platelets and plays an important role in regulating platelet activation/aggregation [45, 46]. *TBXAS1* gene encodes the thromboxane-A synthase 1, an enzyme responsible for the conversion of prostaglandin H2 (PGH2) to thromboxane A2 (TXA2), a potent inducer of blood vessel constriction and platelet aggregation [47]. Data from the literature also evidenced the association of thromboxane A synthase 1 gene expression and promotor haplotypes with the risk of large artery-atherosclerosis stroke in the Iranian population [48]. As concerns *ENTPD1*, it encodes the ectonucleoside triphosphate diphosphohydrolase 1 observed to be implicated in the prevention of platelet aggregation by hydrolyzing platelet-activating ADP to AMP [49]. VITT06 patient also exhibited uncertain significance rare variants in *FFAR2*, *NDST1*, and *NLRP2* genes. *FFAR2* gene encodes a G protein-coupled receptor that is activated by a major product of dietary fiber digestion, the short-chain fatty acids (SCFAs), and that plays a role in the regulation of whole-body energy homeostasis and in intestinal immunity. Previous data from the literature reported its role as a potential biomarker to predict AMI occurrence [50]. NDST1 encodes a member of the heparan sulfate/heparin GlcNAc N-deacetylase/N-sulfotransferase family. It has been previously reported in mouse models that modification of the heparan sulfate fine structure by Ndst1 deletion contributes to decrease vascular smooth muscle cells (VSMC) proliferation, as well as vascular remodeling [51]. NDS1 deficiency has also been previously shown to correlate with allergic airway inflammation [52]. The *NLRP2* gene encodes a member of the nucleotide-binding and leucine-rich repeat receptor (NLR) family. It interacts with components of the IkB kinase (IKK) complex and can regulate both caspase-1 and NF-*κ*B (nuclear factor kappa-light-chain-enhancer of activated B cells) activity. Previous data from the literature reported higher expression levels both in vitro and in vivo under ischemic stroke conditions [53].

In VITT03, in whom a deep vein thrombosis was observed, a missense VUS [c.1228G>T(p.Asp410Tyr)] classified by all in silico predictors as damaging has been identified in *EPB41* gene, encoding for a structural element of the erythrocyte membrane skeleton, for which gene expression studies evidenced its contribution in the thrombus formation network [54]. Actually, a decreased erythrocyte membrane flexibility results in an increased red erythrocyte rigidity that might contribute to a thrombogenic potential [55]. Apart from *EPB41*, VITT03 also carries a further missense VUS in the *PDIA6* gene, encoding for the protein disulfide-isomerase A6, that, beyond its role as a molecular chaperone, able to inhibit aggregation of misfolded proteins, also plays a role in platelet aggregation [56]. VITT03 patient also carries uncertain significance rare variants in *AKT1* and *NOTCH2* genes. *AKT1* gene encodes one of the three members of the human AKT serine-threonine protein kinase family. It has been demonstrated to contribute to dendritic cell survival and maturation [57]. Moreover, *AKT1* mutations are associated with Proteus syndrome, characterized by an increased risk of thromboembolism [58]. *NOTCH2* gene encodes a member of the Notch family, playing a role in a variety of developmental processes by controlling cell fate decisions. Data from the literature reported that anti-PF4/heparin antibody production is impaired in B-cell–specific Notch2-deficient mice, lacking marginal zone (MZ) B cells [59].

In VITT21 VUS in *COL5A1* and *LRTM2* loci have been observed; interestingly, the *LRTM2* gene encoded the leucine-rich repeat and transmembrane domain-containing protein 2 also involved in heparin binding. Moreover, this patient also displayed a further VUS in the *COL5A1* gene, encoding the alpha 1 chain of the type V collagen, able to bind thrombospondin as well as heparin [60, 61], thus supporting its possible involvement in the clinical phenotype. VITT21 also carries a VUS in the *ADAM8* gene. *ADAM8* gene encodes a member of the ADAM (a disintegrin and metalloprotease domain) family, a family of transmembrane glycoproteins with heterogeneous expression profiles and proteolytic, cell-adhesion, -fusion, and -signaling properties. Data from the literature reported that ADAM8 has immunomodulation properties; in particular, it has been observed to be associated with the function of neutrophils during inflammatory responses [62].

In conclusion, WES analysis performed in 6 patients with VITT identifies a number of rare variants which are conditions possibly favoring the prothrombotic state triggered by the exposure to the vaccine.

To the best of our knowledge, no other studies are present in the literature sequencing patients with VITT. Only a recent paper addressed the genetic characteristics of a VITT patient by genotyping more than 870,000 known SNPs in the human genome with an approach not able to identify unknown and rare variants [16].

The observation of a burden of VUS for each VITT patient might raise the issue of whether one or more VUS are causative variants, and whether, instead, we should hypothesize a burden of susceptibility genetic factors in the modulation of the clinical phenotype after an acquired condition, such the exposure to adenovirus.

Data of the present study, to be confirmed and implemented, need functional studies and/or an extension to a larger number of patients, thus allowing a more comprehensive definition of these molecular pathways in modulating clinical manifestations of this condition also in consideration of a possible evaluation of the combined contribution of rare and common variants.

## Figures and Tables

**Table 1:** Demographic and clinical characteristics of the study population.

**Patient**	**Sex**	**Age (years)**	**Vaccine**	**Venous thrombosis**	**Arterial thrombosis**	**Bleeding**	**Clinical outcome**
VITT02	Female	57	Vaxzevria	PE and PVT	Abdominal aorta and lienal artery		No disability
VITT03	Female	78	Janssen	DVT			No disability
VITT05	Female	75	Vaxzevria	PVT and CSVT		SDH, IVH, and IPH	Deceased
VITT06	Female	73	Vaxzevria	CSVT, PE, RVT	Stroke and lower extremities		No disability
VITT18	Female	42	Vaxzevria		Stroke	IPH	Severe disability
VITT21	Female	60	Vaxzevria	DVT, CSVT, SVT, and PE			No disability

Abbreviations: CSVT, cerebral sinuses venous thrombosis; DVT, deep venous thrombosis; IPH, intraparenchymal hemorrhage; IVH, intraventricular hemorrhage; PE, pulmonary embolism; PVT, portal venous thrombosis; RVT, renal venous thrombosis; SDH, subdural hematoma; SVT, splanchnic venous thrombosis.

**Table 2:** Rare uncertain significance variants identified in patients with VITT in the selected genes.

**Patient**	**Gene symbol and transcript ID**	**dbSNP ID**	**Genetic variant nomenclature**	**Minor allele frequency**	**In silico prediction**					**NetGene2**	**ASSP**	**SpliceAI**	**ACGM variant classification**
					**FATHMM**	**MutationTaster**	**Provean**	**Polyphen**	**Sift**				
VITT02	ITGA2B NM_000419	rs199641871	c.457G>A (p.Ala153Thr)	0.0002339	D	D.C.	N	D	T				VUS
VITT02	ITGAD NM_005353	rs147321998	c.736C>T (p.Arg246Ter)	0.002425	—	—	—	—	—				VUS
VITT02	THBD NM_000361	rs888161210	c.1465G>C (p.Asp489His)	N/A	T	P	N	P.D.	D				VUS
VITT02	FGA NM_000508	rs1257998751	c.1132T>A (p.Ser378Thr)	N/A	T	P	N	B	T				VUS
VITT02	CTNNA1 NM_001903	rs371054484	c.377G>C (p.Arg126Pro)	0.00001548	T	D.C.	D	P.D.	D				VUS
VITT02	PIEZO1 NM_001142864	rs776672249	c.2545C>T (p.Arg849Cys)	0.00001668	T	D.C.	D	D	D				VUS
VITT02	GLCE NM_015554	rs199559391	c.1357A>G (p.Thr453Ala)	0.0001583	T	D.C.	N	D	T				VUS
VITT02	ENPP6 NM_153343	rs147979605	c.541C>G (p.Arg181Gly)	0.00001765	T	D.C.	N	B	T				VUS
VITT02	SERPINA12 NM_173850	rs61758960	c.608T>C (p.Leu203Pro)	0.004	D	D.C.	D	D	D				VUS
VITT02	BMPR1B NM_001256793	rs772708128	c.596_598del (p.Tyr199_Ile200delinsPhe)	0.0000177	—	—	—	—	—				VUS
VITT02	CARD8 NM_001184900	rs369625179	c.1217A>G (p.Tyr406Cys)	0.0000439	T	P	D	P.D.	D				VUS
VITT02	EGFR NM_005228	rs371229748	c.3244A>T (p.Ile1082Leu)	0.0000266	T	P	N,D	B	T				VUS
VITT02	JMJD7-PLA2G4B NM_005090	rs758353242	c.1813C>G (p.Leu605Val)	0.00013	T	P	N	D	D				VUS
VITT03	EPB41 NM_001166005	rs150835844	c.1228G>T (p.Asp410Tyr)	0.0002479	D	D.C.	D	D	D				VUS
VITT03	PDIA6 NM_005742	rs768426261	c.256C>T (p.His86Tyr)	0.00007778	T	D.C.	D	D	T				VUS
VITT03	DST NM_001144769	N/A	c.901C>T (p.Pro301Ser)	N/A	D	D.C.	D	—	D				VUS
VITT03	SMPD1 NM_000543	rs142215226	c.340G>A (p.Val114Met)	0.001060	D	D.C.	N	—	D				VUS
VITT03	AKT1 NM_001014431	rs768025881	c.349_351dup (p.Glu117dup)	0.0000088	—	—	—	—	—				VUS
VITT03	NOTCH2 NM_024408	rs765404709	c.7114G>C (p.Ala2372Pro)	0.00000879	D	D.C.	N	B	T				VUS
VITT05	STAB2 NM_017564	rs1030976854	c.2065C>G (p.Pro689Ala)	0.000008793	T	D.C.	D	D	D				VUS
VITT05	CD47 NM_001777	rs369671811	c.311A>G (p.Asp104Gly)	0.00005315	T	P	D	P.D.	D				VUS
VITT05	COL6A1 NM_001848.3	rs398123635	c.2191C>T (p.Arg731Cys)	0.0000562	D	D.C.	D	D	D				VUS
VITT05	IL5RA NM_175726.4	rs145815803	c.913G > A (p.Asp305Asn)	0.0000704	D	P	N	B	T				VUS
VITT06	FGA NM_000508	N/A	c.2068C>G (p.Gln690Glu)	N/A	D	D.C.	N	B	T				VUS
VITT06	VASP NM_003370	rs202205375	c.1130G>C (p.Arg377Pro)	0.00001771	T	D.C.	N	D	D				VUS
VITT06	TBXAS1 NM_001166253	rs759354516	c.1673T>C (p.Leu558Pro)	0.00005285	T	D.C.	D	D	T				VUS
VITT06	PIK3CA NM_006218.4	N/A	c.834G>A (p.Met278Ile)	N/A	T	D.C.	N	B	T				VUS
VITT06	LIMS2 NM_017980	rs770469332	c.254G>A (p.Arg85Gln)	0.0000177	D	D.C.	D	P.D.	D				VUS
VITT06	VCL NM_014000.3	rs754046223	c.1298G>A (p.Arg433His)	N/A	T	D.C.	N	P.D.	D				VUS
VITT06	ENTPD1 NM_001164178	rs142591047	c.1226T>C (p.Ile409Thr)	0.000007742	T	P	D	B	T				VUS
VITT06	FFAR2 NM_005306.3	N/A	c.913G>A (p.Asp305Asn)	N/A	T	P	N	B	T				VUS
VITT06	NDST1 NM_001543.5	rs201660056	c.2426C>T (p.Ala809Val)	0.0000439	D	D.C.	N	B	D				VUS
VITT06	NLRP2 NM_001174081.3	rs140225599	c.398-2A>T	0.000651	—	—	—	—	—	Suggestive of splicing alteration	Suggestive of splicing alteration	Suggestive of splicing alteration	VUS
VITT06	RPS6KA4 NM_003942.3	rs138221123	c.427G>A (p.Gly143Ser)	0.000571	T	D.C.	D	D	D				VUS
VITT18	GP6 NM_001083899	rs779660245	c.830G>A (p.Arg277Gln)	<0.0001	T	P	D	D	D				VUS
VITT18	SERPINA12 NM_173850	rs146053420	c.1062C>G (p.His354Gln)	0.0008485	D	D.C.	D	D	D				VUS
VITT18	MFSD2B NM_001346880	rs138903557	c.1000C>G (p.Pro334Ala)	0.003837	D	D.C.	D	D	D				VUS
VITT18	NID2 NM_007361	N/A	c.2353A>G (p.Thr785Ala)	N/A	D	P	D	B	D				VUS
VITT18	COLGALT1 NM_024656	rs764429704	c.487C>G (p.Leu163Val)	0.00002324	D	D.C.	N	P.D.	D				VUS
VITT18	WDFY4 NM_020945	rs765608663	c.193C>T (p.Arg65Cys)	0.0001555	T	P	D	D	D				VUS
VITT18	SAMD9L NM_152703	rs776780720	c.1076G>A (p.Arg359Gln)	0.00001765	T	P	N	D	D				VUS
VITT18	BCL6 NM_001706	rs1234897185	c.592T>C (p.Tyr198His)	0.00000879	T	D.C.	N	D	T				VUS
VITT18	TRPV1 NM_018727	N/A	c.1064A > G (p.Gln355Arg)	N/A	T	D.C.	N	B	T				VUS
VITT21	LRTM2 NM_001163926	rs919752328	c.1046G>A (p.Arg349His)	N/A	T	D.C.	N	D	D				VUS
VITT21	COL5A1 NM_001278074	rs201997623	c.404C>T (p.Ser135Phe)	0.00001763	T	D.C.	D	D	D				VUS
VITT21	ADAM8 NM_001109.5	rs368130036	c.923C>T (p.Ala308Val)	0.000121	T	P	D,N,D	P.D.	D				VUS
VITT21	JMJD7-PLA2G4B NM_005090.4	rs146887953	c.66G>T (p.Glu22Asp)	0.00103	T	P	N	B	T				VUS

Abbreviations: B, benign variant; D, damaging; DC, disease causing;; LB, likely benign variant; N, neutral; P, polymorphism; PD, possibly/probably damaging; T, tolerated; VUS, variant of uncertain clinical significance.

## Data Availability

Data are available at request by contacting the corresponding author.

## Supporting Information

Supporting Information Additional supporting information can be found online in the Supporting Supplementary Table S1: genes for variant analysis selected according to Gene Ontology biological processes or Human Phenotype Ontology; Supplementary Table S2: rare variants identified in VITT patients in the selected genes.
