# Supplementary material for: Whole Exome Sequencing in Vaccine-Induced Thrombotic Thrombocytopenia (VITT)
Source: Biomed Res Int. 2024 Jul 14;2024:2860547. doi: 10.1155/2024/2860547 (PMC11260508; doi:10.1155/2024/2860547)
Supplement: Supporting Information — Additional supporting information can be found online in the Supporting Supplementary Table S1: genes for variant analysis selected according to Gene Ontology biological processes or Human Phenotype Ontology; Supplementary Table S2: rare variants identified in VITT patients in the selected genes. [file 2860547.f1.docx]

**Supplementary Table 1.** Genes for variants analysis selected according to Gene Ontology biological processes or Human Phenotype Ontology

| **Pathway** | **Pathway ID** | **Genes** |
| --- | --- | --- |
| Blood coagulation | GO:0007596 | *MERTK, SLC7A11, PF4, GP5, ITGB3, DGKA, ANXA5, F7, GAS6, F13B, SERPING1, GNA13, KLKB1, F11, PROCR, LNPK, PDIA3, HNF4A, F2R, F3, PROC, GNA12, ADRA2C, PRSS56, CTSG, SRF, CD59, ADRA2A, ADRA2B, FLNA, CD9, PLCG2, PIK3CG, KNG1, SERPINA1, SERPINC1, GGCX, GP6, VCL, VWF, F2RL1, FBLN1, DGKK, HBB, PAFAH2, METAP1, CSRP1, ANO6, SLC6A4, JMJD1C, CD36, GNAQ, TSPAN32, GP1BB, PLEK, CLIC1, GP9, PLAUR, ILK, RAP2B, HRG, PABPC4, CYP4F11, WNT3A, MYL9, DGKZ, ITPK1, RAB27A, CYP4F2, FGL1, MMRN1, TREML1, HPS6, UBASH3B, PDPN, P2RX1, STXBP1, STXBP3, SHH, LCK, PDGFRA, TFPI2, ACTG1, SYK, DGKH, SCUBE1, F2RL2, HSPB1, ANXA8, MYH9, ENTPD1, FZD6, P2RY12, GP1BA, MAPK14, WAS, F2RL3, GNAS, DGKI, F5, DGKD, SERPIND1, COL3A1, HPS5, VKORC1, CD40LG, APOH, SLC4A1, ADAMTS13, PLSCR1, PDIA2, SRC, CPB2, DGKG, TYRO3, PEAR1, PLAU, F12, F10, F9, F2, MYL12A, HPS4, PTPN6, PIK3CB, BLOC1S4, FGG, FGB, FGA, ACTB, AXL, PAPSS2, BLOC1S6, VAV3, LMAN1, TFPI, DTNBP1, ITGA2, P2RY1, HGFAC, PIK3CA, ENTPD2, ENPP4, VAV2, C4BPB, SERPINE2, PPIA, MPIG6B, DGKB, GATA1, C1GALT1C1, CD40, PROS1, THBD, AK3, SAA1, SERPINA10, PROZ, BLOC1S3, DGKE, PLG, PLAT, COMP, ADORA2A, TUBB1, FIBP, DGKQ, F13A1, F8, TLN1, AP3B1, IL6, VAV1, FERMT3* |
| Fibrinolysis | GO:0042730 | *KRT1, SERPING1, SERPINB2, KLKB1, SERPINF2, HRG, GP1BA, ANXA2, CPB2, PLAU, F12, F2, FGG, FGB, FGA, PROS1, SERPINE1, PLG, PLAT* |
| Integrin mediated signaling pathway | GO:0007229 | *CDH17, ITGB3, PLPP3, ISG15, ITGB2, PTK2B, BCAR1, DOCK1, NRP1, ITGB1BP1, RCC2, ITGA5, HCK, ITGB6, LIMS2, CEACAM1, ITGA4, ADAM10, ITGAE, ITGB5, COL16A1, ITGAM, ITGA8, ZNF304, ITGA9, ITGAV, ZYX, PTN, CD47, CTNNA1, TSPAN32, PLEK, ITGA7, CUL3, ADAM9, THY1, ILK, FGR, ITGAD, DST, ITGB8, ITGB7, ITGA3, FUT8, ITGA2B, MADCAM1, ADAM15, SYK, PRKD1, MYH9, FYB2, LAT, ERBIN, LAMA5, COL3A1, FERMT1, CDC42, CD40LG, FN1, ADAMTS13, FYB1, SRC, PRAM1, ITGA11, ABL1, APOA1, ITGA1, NEDD9, VAV3, CCM2, ITGBL1, ITGA2, ITGAX, ITGAL, TEC, ADAM11, ITGB1, MPIG6B, ANGPTL3, FERMT2, TXK, ITGA10, NME2, ADAMTS1, CCN2, SEMA7A, PTPRA, PTPN11, TLN1, ITGB4, ITGA6, VAV1, FERMT3, PTK2* |
| Autoimmune thrombocytopenia | HP:0001973 | *ACP5, ADA, ARHGEF1, CASP10, CD19, CD81, CIITA, CR2, CTLA4, CTNNBL1, FAS, FASLG, FOXP3, GALC, ICOS, IL7R, IRF2BP2, ITK, KDM6A, KMT2D, KRAS, LAT, LRBA, MAGT1, MS4A1, NFKB1, NFKB2, NRAS, PNP, PRKCD, RASGRP1, RFX5, RFXANK, RFXAP, SASH3, SMPD1, SOCS1, STAT1, STAT3, TLR7, TNFRSF13B, TNFRSF13C, TNFSF12, TOM1, TPP2, ZAP70* |
| Platelet aggregation | GO:0070527 | *SLC7A11, ITGB3, PDIA3, FLNA, PIK3CG, VCL, HBB, METAP1, CSRP1, SLC6A4, TSPAN32, PLEK, CLIC1, ILK, RAP2B, WNT3A, MYL9, FGL1, UBASH3B, STXBP1, STXBP3, PDGFRA, ACTG1, HSPB1, MYH9, P2RY12, F2RL3, GNAS, PDIA2, TYRO3, PEAR1, MYL12A, PTPN6, PIK3CB, BLOC1S4, FGG, FGB, FGA, ACTB, PPIA, GATA1, COMP, TUBB1, FIBP, TLN1, FERMT3* |
| Platelet activation | GO:0030168 | *MERTK, SLC7A11, PF4, ITGB3, DGKA, GNA13, PDIA3, F2R, ADRA2C, CTSG, SRF, ADRA2A, ADRA2B, FLNA, CD9, PLCG2, PIK3CG, GP6, VCL, VWF, DGKK, HBB, METAP1, CSRP1, SLC6A4, TSPAN32, GP1BB, PLEK, CLIC1, ILK, RAP2B, HRG, WNT3A, MYL9, DGKZ, FGL1, TREML1, UBASH3B, PDPN, P2RX1, STXBP1, STXBP3, LCK, PDGFRA, ACTG1, SYK, DGKH, F2RL2, HSPB1, MYH9, FZD6, P2RY12, GP1BA, MAPK14, F2RL3, GNAS, DGKI, DGKD, COL3A1, CD40LG, ADAMTS13, PLSCR1, PDIA2, SRC, DGKG, TYRO3, PEAR1, F2, MYL12A, PTPN6, PIK3CB, BLOC1S4, FGG, FGB, FGA, ACTB, AXL, VAV3, P2RY1, PIK3CA, ENTPD2, VAV2, SERPINE2, PPIA, DGKB, GATA1, C1GALT1C1, CD40, SAA1, BLOC1S3, DGKE, COMP, TUBB1, FIBP, DGKQ, TLN1, IL6, VAV1, FERMT3* |
| Inflammatory response | GO:0006954 | *LBP, ATAT1, IL17F, ACER3, WDR83, CCL3L1, SMO, MYD88, FOS, TIRAP, S100A9, POLB, SELE, CCL22, CMKLR1, KLRG1, LTB4R, CHIA, TICAM1, IL17D, CAMK1D, CUL3, TRIM55, CX3CL1, HLA-DRB1, P2RX1, LOXL3, TOLLIP, FFAR4, IDO1, TNIP1, LGALS9, CCL24, CCRL2, ODAM, GPER1, S1PR3, HMGB2, CCL19, BLNK, IKBKG, MDK, CXCR6, SNAP23, WNT5A, PYDC2, ALOX15, PLGRKT, NLRP10, NOTCH1, PTX3, CXCL11, ALOX5, GGT5, CHI3L1, OLR1, RIPK1, SCNN1B, NRROS, REG3G, PIK3CD, ATRN, CCR6, CCR5, CYBB, CCR4, CCR3, CCL11, HAVCR2, NINJ1, IL20RB, DPEP1, SCN9A, SYK, LY96, SCYL3, TLR1, TNF, SIRT2, RAC1, IFNA2, CNTF, PRKD1, GRN, AGER, SCUBE1, BMPR1B, TFR2, PLA2G4C, RELB, NUPR1, IL34, CERS6, CYP26B1, BPGM, PTGER1, JAM3, CNR2, IL36G, IL36B, IL37, TREM2, PLA2G10, CCL16, HDAC4, TLR3, CCL25, NR3C1, PLA2G3, DHX9, TSPAN2, NLRP3, HSPG2, TNFRSF1A, IL22, NR1H4, HNRNPA0, PTGER4, NLRP2, XCR1, HAMP, TLR9, APOL2, APOL3, THBS1, TREX1, BMP2, TNFAIP6, HK1, LYN, IL5RA, VCAM1, MIF, TFRC, CXCL10, PPBP, CD40LG, ORM1, FN1, C1QA, APCS, CRP, GPR33, HMGB1, IL4R, CRHBP, PLSCR1, CHUK, ORM2, IL13, TLR7, CD68, TMEM258, SPHK1, UNC13D, FPR3, CSF1, HMOX1, TSPAN18, IL1RL1, IL10RB, CXCL1, KDM6B, FKRP, ADAM8, ADCY1, TLR10, PLA2G2A, MEFV, FFAR2, CXCL3, CXCL2, PTGDR, NFKB1, FOLR2, HYAL3, CCL26, PLAA, DAB2IP, C5AR2, ACKR1, CAMK4, IL17A, IL23R, CSF1R, PRCP, SIGIRR, LAT, PSTPIP1, TLR6, RPS6KA4, MMP25, JMJD7-PLA2G4B, IL17RC, EIF2AK1, HRH4, TXNIP, RIPK2, CXCR3, TMIGD3, ADORA3, CASP4, TUSC2, WNK4, FUT7, CXCL13, CHST1, TYROBP, F12, HDAC9, HP, F2, IFI16, HIF1A, COL6A1, CCL14, CELA1, EPHA2, TNIP3, AGTR1, MACIR, LIAS, IRGM, EGFR, RPS6KA5, FASN, KIT, PF4V1, BTK, SAA2, SAA1, REG3A, NDST1, LYZ, CD163, SMPDL3B, IL1RL2, LACC1, SIGLEC1, PLD3, TRIM31, CCL20, TAC4, ELF3, SPINK7, UMOD, LY75, CTNNBIP1, TRIM14, NLRP4, FOSL1, FOSL2, NKG7, ECM1, DHRS7B, CXCL5, CCL1, PARP4, CEBPB, UGT1A1, IL10, CLU, CD5L, AHSG, GPR32, RBPJ, IL25, ASS1, SCYL1, FCGR2A, FCGR1A, AOC3, TNFRSF1B, MEP1B, MYLK3, IL17C, IL1RAP, ADORA2A, IL23A, ITGAL, ACVR1, NFE2L2, PRKCQ, NOTCH2, MTOR, TNIP2, IL33, CD2AP, IL17RE, ADGRE2, ITGB1, IRAK2, FCGR3B, PBXIP1, SCN11A, TRAF3IP2, NAIP, AGR2, FUT4, SLC11A1, MFHAS1, CHST2, CX3CR1, SNCA, CCL4L2, MAPT, IL17B, BMP6, TBK1, NFKBID, IL36A, CCR7, CCR1, STK39, REL, FADS2, NFATC4, MAP3K7, C4B, C4A, IL31RA, VNN1, C3AR1, LARGE1, ZEB2, PNMA1, CCL3, CXCL8, CD40, C2CD4A, ZC3H12A, ITIH4, H2BC1, TAC1, ZNF580, PSEN1, FCER1A, CHST4, GNAT2, CYSLTR1, SLAMF8, NOD1, CCL15, GSDMD, PLD4, IL27, NFAM1, MAPKAPK2, SEMA7A, PRDX2, CCL4, CD14, TLR4, IL1R1, S100A12, PLA2G2E, BDKRB1, AP3B1, F8, EXT1, PYCARD, CDO1, TLR5, TLR2, IL18R1, CCR2, IL17RA, ADORA1, CD96, SAA4, NCR3, IKBKB, CSRP3, MAP2K3, PF4, HDAC5, C2CD4B, ADCY8, AIM2, CD44, FFAR3, HYAL1, PJA2, GATA3, AGTR2, CYBA, SLAMF1, KRT16, PLP1, STAB1, JAK2, NOX4, IL5, IL4, S100A8, ITGB2, NLRC4, KLKB1, APP, NLRP1, CD6, IRF5, CLEC7A, BCL6, NFKB2, PLA2G2D, MRGPRX1, B4GALT1, NAGLU, MSMP, ASH1L, IFNGR1, IL9, MAPK9, F2R, F3, LXN, PTAFR, TACR1, CXCR4, HRH1, ANXA1, ADM, GBP5, NFKBIZ, NDP, CXCL9, AFAP1L2, TICAM2, LY86, SELP, ITGB6, IL1RN, KDM4D, SERPINF2, VPS54, FCGR3A, HCK, TNFSF4, TCIRG1, PXK, IL2RA, EPO, IL1B, IL1A, IFNG, FOXP3, ELANE, CARD8, F2RL1, CCL21, ACKR2, AIF1, IL18, NFX1, NFATC3, AIMP1, PRDX5, TGFB1, TMED2, P2RX7, LIPA, KCNJ8, CXCL6, SLC18A2, NLRP9, CCL17, PROK2, TNFAIP3, PIK3CG, KNG1, TIMP1, C5, C3, SERPINA3, SERPINA1, PRKCZ, IFNGR2, MGLL, AKT1, TRIL, PTGER2, PTGER3, SMAD1, MBL2, CCL7, CCL8, ABCF1, INS, TNFRSF4, TLR8, CRH, ADGRE5, TBXA2R, C5AR1, CALCA, CCL13, THEMIS2, FCGR2B, CD180, PARK7, PTN, POMGNT1, ANO6, TRPV1, ITGAM, PYDC5, GPR68, FCGR2C, FPR1, XCL1, NLRP6, IL1F10, RARRES2, PTGFR, LRP1, FPR2, SETD4, CXCR2, ACOD1, CCL18, CCL23, NOS2, PTGIR, A2M, NFKBIB, F11R, CD47, NOX1, CD36, IL6R, SCG2, CCL5, CCL2, CARD18, AXL, IL36RN, TAB2, XCL2, MAP3K20, STAT3, ALOX5AP, POMT2, ITCH, NKIRAS2, RELA, RASGRP1, IL18RAP, BDKRB2, NR4A1, IL6, AZU1* |

**Supplementary Table 2.** Rare variants identified in VITT patients in the selected genes

|  |  |  |  |  | ***In Silico* Prediction** | | | | | | | |  |
| --- | --- | --- | --- | --- | --- | --- | --- | --- | --- | --- | --- | --- | --- |
| **Patient** | **Gene Symbol**  **and**  **Transcript ID** | **dbSNP ID** | **Genetic Variant Nomenclature** | **Minor Allele Frequency** | **FATHMM** | **MutationTaster** | **Provean** | **Polyphen** | **Sift** | **NetGene2** | **ASSP** | **SpliceAI** | **ACGM Variant Classification** |
| **VITT02** | **ITGA2B**  **NM_000419** | **rs199641871** | **c.457G>A (p.Ala153Thr)** | **0.0002339** | **D** | **D.C.** | **N** | **D** | **T** |  |  |  | **VUS** |
| **VITT02** | **ITGAD**  **NM_005353** | **rs147321998** | **c.736C>T (p.Arg246Ter)** | **0.002425** | **-** | **-** | **-** | **-** | **-** |  |  |  | **VUS** |
| **VITT02** | **THBD**  **NM_000361** | **rs888161210** | **c.1465G>C (p.Asp489His)** | **N/A** | **T** | **P** | **N** | **P.D.** | **D** |  |  |  | **VUS** |
| **VITT02** | **FGA**  **NM_000508** | **rs1257998751** | **c.1132T>A (p.Ser378Thr)** | **N/A** | **T** | **P** | **N** | **B** | **T** |  |  |  | **VUS** |
| **VITT02** | **CTNNA1**  **NM_001903** | **rs371054484** | **c.377G>C (p.Arg126Pro)** | **0.00001548** | **T** | **D.C.** | **D** | **P.D.** | **D** |  |  |  | **VUS** |
| **VITT02** | **PIEZO1**  **NM_001142864** | **rs776672249** | **c.2545C>T (p.Arg849Cys)** | **0.00001668** | **T** | **D.C.** | **D** | **D** | **D** |  |  |  | **VUS** |
| **VITT02** | **GLCE**  **NM_015554** | **rs199559391** | **c.1357A>G (p.Thr453Ala)** | **0.0001583** | **T** | **D.C.** | **N** | **D** | **T** |  |  |  | **VUS** |
| **VITT02** | **ENPP6**  **NM_153343** | **rs147979605** | **c.541C>G (p.Arg181Gly)** | **0.00001765** | **T** | **D.C.** | **N** | **B** | **T** |  |  |  | **VUS** |
| **VITT02** | **SERPINA12 NM_173850** | **rs61758960** | **c.608T>C (p.Leu203Pro)** | **0.004** | **D** | **D.C.** | **D** | **D** | **D** |  |  |  | **VUS** |
| **VITT02** | **BMPR1B NM_001256793** | **rs772708128** | **c.596_598del (p.Tyr199_Ile200delinsPhe)** | **0.0000177** | **-** | **-** | **-** | **-** | **-** |  |  |  | **VUS** |
| **VITT02** | **CARD8 NM_001184900** | **rs369625179** | **c.1217A>G (p.Tyr406Cys)** | **0.0000439** | **T** | **P** | **D** | **P.D.** | **D** |  |  |  | **VUS** |
| **VITT02** | **EGFR NM_005228** | **rs371229748** | **c.3244A>T (p.Ile1082Leu)** | **0.0000266** | **T** | **P** | **N,D** | **B** | **T** |  |  |  | **VUS** |
| **VITT02** | **JMJD7-PLA2G4B NM_005090** | **rs758353242** | **c.1813C>G (p.Leu605Val)** | **0.00013** | **T** | **P** | **N** | **D** | **P.D.** |  |  |  | **VUS** |
| VITT02 | SERPINB4 NM_002974 | rs137899169 | c.917A>G (p.Asn306Ser) | 0.0003412 | D | P | N | B | T |  |  |  | LB |
| VITT02 | LRBA  NM_006726 | rs143625481 | c.787C>G (p.Leu263Val) | 0.0009014 | T | D.C. | N | D | T |  |  |  | LB |
| VITT02 | FGB  NM_005141 | rs201909029 | c.534G>C (p.Lys178Asn) | 0.0005977 | D | D.C. | N | B | T |  |  |  | LB |
| VITT02 | HPS5  NM_181507 | rs61755718 | c.3045G>A (p.Met1015Ile) | 0.00798 | T | P | N | B | T |  |  |  | LB |
| VITT02 | PTGER3 NM_198718 | rs568967213 | c.1185del(p.Asn395LysfsTer) | 0.008279 | - | - | - | - | - |  |  |  | LB |
| VITT02 | FANCL NM_001114636 | rs55849827 | c.112C>T (p.Leu38Phe) | 0.004304 | T | D.C. | N | D | D |  |  |  | LB |
| VITT02 | SLX4  NM_032444 | rs150712805 | c.2305G>C (p.Glu769Gln) | 0.001644 | T | P | N | B | T |  |  |  | LB |
| VITT02 | PRDM16  NM_022114 | rs199968728 | c.142G>A (p.Val48Met) | 0.0004263 | T | P | N | B | T |  |  |  | LB |
| VITT02 | TNFSF12  NM_172089.4 | rs768061768 | c.43G>A (p.Glu15Lys) | 0.0002358 | D | P | N | B | D |  |  |  | LB |
| VITT02 | ITGA9  NM_002207 | rs61751189 | c.1691G>A (p.Arg564Gln) | 0.005522 | T | D.C. | N | P.D. | D |  |  |  | LB |
| VITT02 | ITGA11  NM_001004439 | rs568152331 | c.3359G>A (p.Arg1120His) | 0.0002078 | T | P | N | B | T |  |  |  | LB |
| VITT02 | MASTL NM_001172303 | rs36121140 | c.1010C>A (p.Thr337Lys) | 0.001710 | T | P | N | B | T |  |  |  | B |
| VITT02 | PLA2G4A NM_024420 | rs28395831 | c.1909A>G (p.Ile637Val) | 0.009599 | T | D.C. | N | B | T |  |  |  | B |
| VITT02 | BDKRB1 NM_000710 | rs143823168 | c.571C>G (p.Leu191Val) | 0.00318 | T | P | N | D | D |  |  |  | LB |
| VITT02 | FASN NM_004104 | rs2228306 | c.6014T>C (p.Val2005Ala) | 0.00206 | T | D.C. | N | B | T |  |  |  | B |
| VITT02 | FASN NM_004104 | rs9898060 | c.6012-12G>C | 0.00204 | - | - | - | - | - | NEG | NEG | NEG | B |
| VITT02 | FASN NM_004104 | rs17848927 | c.1493-16G>A | 0.00222 | - | - | - | - | - | NEG | NEG | NEG | B |
| VITT02 | TRIM31 NM_007028 | rs201869087 | c.513+3A>G | 0.00387 | - | - | - | - | - | NEG | NEG | DONOR LOSS | B |
| VITT02 | ALOX15 NM_001140 | rs41432647 | c.1849C>T (p.Pro617Ser) | 0.00437 | T | P | N | B | T |  |  |  | LB |
| VITT02 | APOL3 NM_145640 | rs142057520 | c.682G>A (p.Ala228Thr) | 0.00353 | T | P | N | P.D. | T |  |  |  | LB |
| VITT02 | CCL4L2 ENST00000394465 | rs1597769113 | c.208G>A (p.Gly70Ser) | 0.00594 | - | - | - | - | - |  |  |  | LB |
| VITT02 | FASN NM_004104 | rs368725295 | c.6163+8C>G | 0.0000629 | - | - | - | - | - | NEG | NEG | NEG | LB |
| VITT02 | PTGER3 NM_198718 | rs568967213 | c.1185del (p.Asn395LysfsTer9) | 0.00834 | - | - | - | - | - |  |  |  | LB |
| VITT02 | SETD4 NM_017438 | rs140814445 | c.1212A>T (p.Lys404Asn) | 0.00475 | T | P | N | B | T |  |  |  | LB |
| VITT02 | HSPG2 NM_005529 | rs141280063 | c.12874G>A (p.Glu4292Lys) | 0.000967 | T | D.C. | N,D | P.D. | T |  |  |  | LB |
| **VITT03** | **EPB41 NM_001166005** | **rs150835844** | **c.1228G>T (p.Asp410Tyr)** | **0.0002479** | **D** | **D.C.** | **D** | **D** | **D** |  |  |  | **VUS** |
| **VITT03** | **PDIA6**  **NM_005742** | **rs768426261** | **c.256C>T (p.His86Tyr)** | **0.00007778** | **T** | **D.C.** | **D** | **D** | **T** |  |  |  | **VUS** |
| **VITT03** | **DST**  **NM_001144769** | **N/A** | **c.901C>T (p.Pro301Ser)** | **N/A** | **D** | **D.C.** | **D** | **-** | **D** |  |  |  | **VUS** |
| **VITT03** | **SMPD1**  **NM_000543** | **rs142215226** | **c.340G>A (p.Val114Met)** | **0.001060** | **D** | **D.C.** | **N** | **-** | **D** |  |  |  | **VUS** |
| **VITT03** | **AKT1 NM_001014431** | **rs768025881** | **c.349_351dup (p.Glu117dup)** | **0.0000088** | **-** | **-** | **-** | **-** | **-** |  |  |  | **VUS** |
| **VITT03** | **NOTCH2 NM_024408** | **rs765404709** | **c.7114G>C (p.Ala2372Pro)** | **0.00000879** | **D** | **D** | **N** | **B** | **T** |  |  |  | **VUS** |
| VITT03 | PTAFR  NM_000952 | rs138629813 | c.341A>G (p.Asn114Ser) | 0.003554 | T | D.C. | N | P.D. | D |  |  |  | LB |
| VITT03 | SLX4  NM_032444 | rs145137472 | c.2681T>G (p.Val894Gly) | 0.0006039 | T | P | N | B | T |  |  |  | LB |
| VITT03 | PRDM16 NM_022114 | rs371654192 | c.2468G>C (p.Arg823Pro) | 0.004107 | T | D.C. | D | D | D |  |  |  | LB |
| VITT03 | RPL5  NM_000969 | rs11540832 | c.629A>G (p.Tyr210Cys) | 0.008106 | T | D.C. | N | B | T |  |  |  | LB |
| VITT03 | F13B  NM_001994 | rs17514281 | c.1025T>C (p.Ile342Thr) | 0.01049 | T | P | D | P.D. | D |  |  |  | LB |
| VITT03 | HPSE  NM_001098540 | rs61755719 | c.707T>G (p.Phe236Cys) | 0.007042 | T | P | N | B | T |  |  |  | LB |
| VITT03 | SH2B3  NM_005475 | rs183913232 | c.557G>T (p.Ser186Ile) | 0.008390 | T | P | N | B | T |  |  |  | LB |
| VITT03 | DGKB  NM_145695 | rs186133720 | c.2110A>G (p.Lys704Glu) | 0.001064 | T | D.C. | N | B | D |  |  |  | LB |
| VITT03 | PRDM16  NM_022114 | rs201199516 | 843C>G (p.His281Gln) | N/A | D | D.C. | N | P.D. | N |  |  |  | LB |
| VITT03 | RPL5  NM_000969 | rs11540832 | c.629A>G (p.Tyr210Cys) | 0.008106 | T | D.C. | N | B | T |  |  |  | LB |
| VITT03 | LAMA5  NM_005560 | rs527650945 | c.6165+6del | 0.002401 | - | - | - | - | - | DONOR GAIN | DONOR GAIN | NEG | LB |
| VITT03 | ADAM8 NM_001109 | rs36054052 | c.1228G>A (p.Val410Met) | 0.00104 | T | P | N | D | D |  |  |  | LB |
| VITT03 | PTAFR NM_000952 | rs138629813 | c.341A>G (p.Asn114Ser) | 0.00366 | T | D.C. | N | P.D. | D |  |  |  | LB |
| VITT03 | CYBB NM_000397 | rs782223666 | c.675-4C>T | 0.000108 | - | - | - | - | - | NEG | NEG | NEG | B |
| VITT03 | TRIM31 NM_007028 | rs201869087 | c.513+3A>G | 0.00387 | - | - | - | - | - | NEG | NEG | DONOR LOSS | B |
| VITT03 | JAM3 NM_032801 | rs202130887 | c.143-8C>T | 0.000721 | - | - | - | - | - | NEG | NEG | NEG | LB |
| VITT03 | PARP4 NM_006437 | rs61741951 | c.4406C>G (p.Ala1469Gly) | 0.00524 | T | P | N | B | T |  |  |  | LB |
| VITT03 | TRAF3IP2 NM_147686 | rs139282334 | c.649C>A (p.Pro217Thr) | 0.0088 | T | D.C. | N | D | D |  |  |  | LB |
| VITT03 | SCNN1B NM_000336 | rs35731153 | c.245C>G (p.Ser82Cys) | 0.0071 | T | D.C. | D | D | D |  |  |  | LB |
| **VITT05** | **STAB2**  **NM_017564** | **rs1030976854** | **c.2065C>G (p.Pro689Ala)** | **0.000008793** | **T** | **D.C.** | **D** | **D** | **D** |  |  |  | **VUS** |
| **VITT05** | **CD47**  **NM_001777** | **rs369671811** | **c.311A>G (p.Asp104Gly)** | **0.00005315** | **T** | **P** | **D** | **P.D.** | **D** |  |  |  | **VUS** |
| **VITT05** | **COL6A1 NM_001848.3** | **rs398123635** | **c.2191C>T (p.Arg731Cys)** | **0.0000562** | **D** | **D.C.** | **D** | **D** | **D** |  |  |  | **VUS** |
| **VITT05** | **IL5RA NM_175726.4** | **rs145815803** | **c.913G>A (p.Asp305Asn)** | **0.0000704** | **D** | **P** | **N** | **B** | **T** |  |  |  | **VUS** |
| VITT05 | DGKD  NM_152879 | rs139085833 | c.3541C>T (p.Arg1181Trp) | 0.0008063 | D | D.C. | D | D | D |  |  |  | LB |
| VITT05 | CD9  NM_001769 | rs35799798 | c.419G>A (p.Arg140Gln) | 0.0004568 | T | D.C. | N | P.D | T |  |  |  | LB |
| VITT05 | LAMA5  NM_005560 | rs770735145 | c.7146G>T (p.Met2382Ile) | 0.00005385 | T | D.C. | N | P.D. | T |  |  |  | LB |
| VITT05 | BRIP1  NM_032043 | rs878855154 | c.3262C>T (p.His1088Tyr) | 0.000008815 | T | P | N | B | T |  |  |  | LB |
| VITT05 | NISCH  NM_007184 | rs148320456 | c.787T>A (p.Ser263Thr) | 0.0004258 | T | P | N | P.D. | T |  |  |  | LB |
| VITT05 | SERPINB2 NM_002575 | rs138446596 | c.738A>G (p.Ile246Met) | 0.002065 | T | D.C. | N | P.D. | T |  |  |  | LB |
| VITT05 | GP9  NM_000174 | rs202229101 | c.368C>T (p.Pro123Leu) | 0.001617 | T | P | N | B | D |  |  |  | LB |
| VITT05 | HABP2  NM_004132 | rs78201625 | c.364C>T (p.Arg122Trp) | 0.003043 | T | D.C. | N | D | T |  |  |  | LB |
| VITT05 | F2RL3  NM_003950. | rs148865185 | c.656G>A (p.Arg219Gln) | 0.0003117 | T | P | N | B | T |  |  |  | LB |
| VITT05 | PIEZO1 NM_001142864 | rs530486445 | c.1495G>A (p.Val499Ile) | 0.0009186 | D | P | N | B | T |  |  |  | LB |
| VITT05 | ITGB4  NM_000213 | rs149659118 | c.2009G>A (p.Arg670His) | 0.001433 | D | P | N | B | T |  |  |  | LB |
| VITT05 | PRAM1  NM_032152 | rs138042924 | c.1159T>C (p.Ser387Pro) | 0.004513 | T | P | D | - | D |  |  |  | LB |
| VITT05 | HABP2  NM_004132 | rs78201625 | c.364C>T (p.Arg122Trp) | 0.003043 | T | P | N | D | T |  |  |  | B |
| VITT05 | IL6  NM_000600 | rs2069860 | c.485A>T (p.Asp162Val) | 0.007710 | T | P | D | B | N |  |  |  | B |
| VITT05 | VEGFB  NM_003377 | rs111555072 | c.286C>G (p.Gln96Glu) | 0.001883 | T | P | N | P.D. | D |  |  |  | B |
| VITT05 | NFKB1  NM_003998 | rs4648072 | c.1519A>G (p.Met507Val) | 0.007642 | T | P | N | B | T |  |  |  | B |
| VITT05 | DAB2IP NM_032552.4 | rs570101345 | c.2948C>T (p.Ala983Val) | 0.0000266 | T | P | N | B | T |  |  |  | LB |
| VITT05 | GPER1 NM_001098201.3 | rs138657567 | "c.1119T>G (p.Ser373Arg) | 0.00648 | T | P | N | B | D |  |  |  | LB |
| VITT05 | CSF1 NM_000757.6 | rs2229165 | c.1312G>A (p.Gly438Arg) | 0.00696 | T | P | N | B | T |  |  |  | B |
| VITT05 | MTOR NM_004958.4 | rs56412200 | c.7367-3T>C | 0.000651 | - | - | - | - | - | NEG | NEG | NEG | B |
| VITT05 | AGER NM_001206929.2 | rs3176931 | c.1154G>A (p.Arg385Gln) | 0.00189 | T | P | N | B | T |  |  |  | LB |
| VITT05 | AKT1 NM_001014431.2 | rs138386095 | c.288-14C>T | 0.000897 | - | - | - | - | - | NEG | NEG | NEG | LB |
| VITT05 | CHIA NM_201653.4 | rs182022651 | c.169G>A (p.Ala57Thr) | 0.000415 | T | D.C. | D | D | D |  |  |  | LB |
| VITT05 | IL1R1 NM_000877.4 | rs56081302 | c.487-6A>G | 0.00211 | - | - | - | - | - | NEG | NEG | NEG | LB |
| VITT05 | IL1RL1 NM_016232.5 | rs34210856 | c.239C>T (p.Ala80Val) | 0.00215 | T | P | N | B | T |  |  |  | LB |
| VITT05 | IL1RL1 NM_016232.5 | rs34225180 | c.526G>A (p.Ala176Thr) | 0.00212 | T | P | N | B | T |  |  |  | LB |
| VITT05 | NOTCH2 NM_024408.4 | rs41313282 | c.4238T>A (p.Leu1413His) | 0.00515 | T | P | D | B | D |  |  |  | LB |
| VITT05 | IL1R1 NM_000877.4 | rs34889382 | c.604T>C (p.Tyr202His) | 0.0021 | T | P | N | B | T |  |  |  | LB |
| VITT05 | IL1R1 NM_000877.4 | rs34835752 | c.1192G>A (p.Gly398Arg) | 0.00211 | T | P | D | D | D |  |  |  | LB |
| VITT05 | SELE NM_000450.2 | rs73041408 | c.1165C>T (p.Arg389Cys) | 0.0000882 | T | P | N,D | P.D. | D |  |  |  | LB |
| VITT05 | TRIM31 NM_007028.5 | rs142316069 | c.899A>T (p.Asp300Val) | 0.000515 | T | P | D | P.D. | D |  |  |  | LB |
| VITT05 | CD96 NM_005816.5 | rs370809674 | c.1250-13T>A | 0.0000352 | - | - | - | - | - | NEG | NEG | NEG | LB |
| VITT05 | PRDX2 NM_005809.6 | rs778577772 | c.380+5T>C | 0.0000176 | - | - | - | - | - | NEG | NEG | NEG | LB |
| **VITT06** | **FGA**  **NM_000508** | **N/A** | **c.2068C>G (p.Gln690Glu)** | **N/A** | **D** | **D.C.** | **N** | **B** | **T** |  |  |  | **VUS** |
| **VITT06** | **VASP**  **NM_003370** | **rs202205375** | **c.1130G>C (p.Arg377Pro)** | **0.00001771** | **T** | **D.C.** | **N** | **D** | **D** |  |  |  | **VUS** |
| **VITT06** | **TBXAS1**  **NM_001166253** | **rs759354516** | **c.1673T>C (p.Leu558Pro)** | **0.00005285** | **T** | **D.C.** | **D** | **D** | **T** |  |  |  | **VUS** |
| **VITT06** | **PIK3CA**  **NM_006218.4** | **N/A** | **c.834G>A (p.Met278Ile)** | **N/A** | **T** | **D.C.** | **N** | **B** | **T** |  |  |  | **VUS** |
| **VITT06** | **LIMS2**  **NM_017980** | **rs770469332** | **c.254G>A (p.Arg85Gln)** | **0.0000177** | **D** | **D.C.** | **D** | **P.D.** | **D** |  |  |  | **VUS** |
| **VITT06** | **VCL**  **NM_014000.3** | **rs754046223** | **c.1298G>A (p.Arg433His)** | **N/A** | **T** | **D.C.** | **N** | **P.D.** | **D** |  |  |  | **VUS** |
| **VITT06** | **ENTPD1 NM_001164178** | **rs142591047** | **c.1226T>C (p.Ile409Thr)** | **0.000007742** | **T** | P | **D** | **B** | **T** |  |  |  | **VUS** |
| **VITT06** | **FFAR2 NM_005306.3** | **N/A** | **c.913G>A (p.Asp305Asn)** | **N/A** | **T** | **P** | **N** | **B** | **T** |  |  |  | **VUS** |
| **VITT06** | **NDST1 NM_001543.5** | **rs201660056** | **c.2426C>T (p.Ala809Val)** | **0.0000439** | **D** | **D.C** | **N** | **B** | **D** |  |  |  | **VUS** |
| **VITT06** | **NLRP2 NM_001174081.3** | **rs140225599** | **c.398-2A>T** | **0.000651** | **-** | **-** | **-** | **-** | **-** | **Suggestive of splicing alteration** | **Suggestive of splicing alteration** | **Suggestive of splicing alteration** | **VUS** |
| **VITT06** | **RPS6KA4 NM_003942.3** | **rs138221123** | **c.427G>A (p.Gly143Ser)** | **0.000571** | **T** | **D.C** | **D** | **D** | **D** |  |  |  | **VUS** |
| VITT06 | GAS6  NM_000820 | rs770488795 | c.1592G>A (p.Arg531His) | 0.0001482 | T | P | N | B | T |  |  |  | LB |
| VITT06 | SERPINA10 NM_016186 | rs2232708 | c.811G>A (p.Gly271Ser) | 0.006813 | D | P | N | B | T |  |  |  | LB |
| VITT06 | LRRFIP1 NM_001137552 | rs3739041 | c.1897A>G (p.Lys633Glu) | 0.003172 | T | P | N | B | T |  |  |  | LB |
| VITT06 | LRRFIP1 NM_001137552 | rs3739040 | c.1934C>T (p.Pro645Leu) | 0.003196 | T | P | N | P.D. | T |  |  |  | LB |
| VITT06 | SLX4  NM_032444 | rs147492092 | c.2654C>T (p.Pro885Leu) | 0.0004568 | T | P | D | P.D. | D |  |  |  | LB |
| VITT06 | UNC13D NM_199242 | rs118049905 | c.2896C>T (p.Arg966Trp) | 0.007380 | T | D.C. | D | D | D |  |  |  | LB |
| VITT06 | COL5A1 NM_001278074 | rs761079177 | c.805G>A (p.Glu269Lys) | 0.0001084 | D | D.C. | N | B | T |  |  |  | LB |
| VITT06 | ENTPD2 NM_203468 | rs374256982 | c.980G>A (p.Arg327Gln) | 0.00003122 | T | P | N | B | T |  |  |  | LB |
| VITT06 | PIEZO1  NM_001142864 | rs564220264 | c.4526G>A (p.Ser1509Asn) | 0.00003554 | T | P | N | B | T |  |  |  | LB |
| VITT06 | RFX5  NM_000449.4 | rs1374291718 | c.1283A>G (p.Lys428Arg) | N/A | T | D.C. | N | D | D |  |  |  | LB |
| VITT06 | PPAN-P2RY11  NM_001040664 | rs199800140 | c.1018C>T (p.Arg340Trp) | 0.0003402 | T | D.C. | D | D | D |  |  |  | LB |
| VITT06 | PPAN-P2RY11  NM_001040664 | rs776329867 | c.1730C>T (p.Ala577Val) | 0.000009221 | T | P | N | B | T |  |  |  | LB |
| VITT06 | SERPINA10  NM_016186.2 | rs2232698 | c.262C>T (p.Arg88Ter) | 0.006544 | - | - | - | - | - |  |  |  | B |
| VITT06 | LRRFIP1  NM_001137552 | rs3739041 | c.1897A>G (p.Lys633Glu) | 0.003172 | T | P | N | B | T |  |  |  | B |
| VITT06 | LRRFIP1  NM_001137552 | rs3739040 | c.1934C>T (p.Pro645Leu) | 0.003196 | T | P | N | B | T |  |  |  | B |
| VITT06 | KMT2D NM_003482 | rs189888707 | c.7670C>T (p.Pro2557Leu) | 0.006668 | T | D.C. | D | B | D |  |  |  | B |
| VITT06 | SELE NM_000450.2 | rs79478039 | c.975C>A (p.Phe325Leu) | 0.00423 | T | P | D | B | T |  |  |  | LB |
| VITT06 | ABCF1 NM_001025091.2 | rs6902544 | c.592A>G (p.Asn198Asp) | 0.00822 | T | P | N | B | T |  |  |  | LB |
| VITT06 | KDM4D NM_018039.3 | rs76057256 | c.1070A>G (p.Glu357Gly) | 0.00202 | T | P | D | D | D |  |  |  | LB |
| VITT06 | NLRP9 NM_176820.4 | rs139574367 | c.2331-17G>A | 0.00385 | - | - | - | - | - | NEG | NEG | NEG | LB |
| VITT06 | LGAL59 NM_009587.3 | rs61736863 | c.675G>T (p.Met225Ile) | 0.00449 | T | P | N | B | T |  |  |  | LB |
| VITT06 | UNC13D NM_199242.3 | rs118049905 | c.2896C>T (p.Arg966Trp) | 0.00763 | T | D.C | D | D | D |  |  |  | LB |
| **VITT18** | **GP6**  **NM_001083899** | **rs779660245** | **c.830G>A (p.Arg277Gln)** | **0.00005**  **(ALFA_EU)** | **T** | **P** | **D** | **D** | **D** |  |  |  | **VUS** |
| **VITT18** | **SERPINA12 NM_173850** | **rs146053420** | **c.1062C>G (p.His354Gln)** | **0.0008485** | **D** | **D.C.** | **D** | **D** | **D** |  |  |  | **VUS** |
| **VITT18** | **MFSD2B NM_001346880** | **rs138903557** | **c.1000C>G (p.Pro334Ala)** | **0.003837** | **D** | **D.C.** | **D** | **D** | **D** |  |  |  | **VUS** |
| **VITT18** | **NID2**  **NM_007361** | **N/A** | **c.2353A>G (p.Thr785Ala)** | **N/A** | **D** | **P** | **D** | **B** | **D** |  |  |  | **VUS** |
| **VITT18** | **COLGALT1 NM_024656** | **rs764429704** | **c.487C>G (p.Leu163Val)** | **0.00002324** | **D** | **D.C.** | **N** | **P.D.** | **D** |  |  |  | **VUS** |
| **VITT18** | **WDFY4**  **NM_020945** | **rs765608663** | **c.193C>T (p.Arg65Cys)** | **0.0001555** | **T** | **P** | **D** | **D** | **D** |  |  |  | **VUS** |
| **VITT18** | **SAMD9L**  **NM_152703** | **rs776780720** | **c.1076G>A (p.Arg359Gln)** | **0.00001765** | **T** | **P** | **N** | **D** | **D** |  |  |  | **VUS** |
| **VITT18** | **BCL6 NM_001706** | **rs1234897185** | **c.592T>C (p.Tyr198His)** | **0.00000879** | **T** | **D.C.** | **N** | **D** | **T** |  |  |  | **VUS** |
| **VITT18** | **TRPV1 NM_018727** | **N/A** | **c.1064A>G (p.Gln355Arg)** | **N/A** | **T** | **D.C.** | **N** | **B** | **T** |  |  |  | **VUS** |
| VITT18 | PAFAH1B3 NM_001145940 | rs141406696 | c.395G>A (p.Arg132Gln) | 0.00005419 | T | P | N | B | T |  |  |  | LB |
| VITT18 | NOS3  NM_000603 | rs3918234 | c.2945A>T (p.Gln982Leu) | 0.002383 | T | D.C. | D | B | T |  |  |  | LB |
| VITT18 | TYRO3  NM_006293 | rs752121582 | c.550G>A  (p.Ala184Thr) | 0.00006219 | T | P | N | B | T |  |  |  | LB |
| VITT18 | MMRN1  NM_007351 | rs147451161 | c.3680G>T (p.Arg1227Leu) | 0.002155 | D | D.C. | D | D | D |  |  |  | LB |
| VITT18 | FYB  NM_001243093 | rs201568060 | c.440C>T (p.Pro147Leu) | 0.001052 | T | P | D | B | T |  |  |  | LB |
| VITT18 | MYH9  NM_002473 | rs139134727 | c.4396C>T (p.Arg1466Trp) | 0.003004 | T | D.C. | D | D | D |  |  |  | LB |
| VITT18 | ADAM17  NM_003183 | rs61754178 | c.148A>G (p.Ile50Val) | 0.005523 | T | D.C. | N | D | T |  |  |  | LB |
| VITT18 | MB21D1/CGAS  NM_138441 | rs147035222 | c.50C>G (p.Ala17Gly) | 0.006627 | T | P | N | B | T |  |  |  | LB |
| VITT18 | PTGS1  NM_000962 | rs5794 | c.1441G>A (p.Val481Ile) | 0.009401 | T | P | N | B | D |  |  |  | LB |
| VITT18 | BRCA2  NM_000059 | rs41293475 | c.1151C>T (p.Ser384Phe) | 0.001188 | T | P | N | D | D |  |  |  | LB |
| VITT18 | ALOX15B NM_001141 | rs141549870 | c.1574G>A (p.Ser525Asn) | 0.0004265 | T | P | N | B | T |  |  |  | LB |
| VITT18 | HPS4  NM_152841 | rs147435410 | c.1396C>T (p.Arg466Cys) | 0.002767 | T | P | N | B | T |  |  |  | LB |
| VITT18 | DGKG  NM_001346 | rs762508343 | c.1684G>A (p.Val562Ile) | 0.00000882 | D | D.C. | N | B | T |  |  |  | LB |
| VITT18 | SCUBE1  NM_173050.5 | rs144373347 | c.2449G>A (p.Asp817Asn) | 0.004394 | T | D.C. | N | B | T |  |  |  | LB |
| VITT18 | ITLN1  NM_017625.3 | rs150008974 | c.509G>A (p.Arg170His) | 0.0002408 | T | P | N | B | T |  |  |  | LB |
| VITT18 | MB21D1/CGAS  NM_13844 | rs147035222 | c.50C>G (p.Ala17Gly) | 0.006627 | T | P | N | B | T |  |  |  | LB |
| VITT18 | LRRFIP1  NM_001137552 | rs142698219 | c.2201C>T (p.Pro734Leu) | 0.001077 | T | P | N | B | T |  |  |  | LB |
| VITT18 | ITGAX  NM_000887 | rs200451726 | c.1798G>A (p.Val600Met) | 0.0004921 | T | P | N | P.D. | T |  |  |  | LB |
| VITT18 | PLCG2  NM_002661.5 | rs75472618 | c.1712A>G (p.Asn571Ser) | 0.007328 | D | D.C. | N | B | D |  |  |  | B |
| VITT18 | C5 NM_001735 | rs142459293 | c.4204A>G (p.Ile1402Val) | 0.000897 | T | P | N | B | D |  |  |  | LB |
| VITT18 | CCR5 NM_001100168 | rs1800940 | c.180G>T (p.Arg60Ser) | 0.00157 | T | P | D | B | D |  |  |  | LB |
| VITT18 | PYDC2 NM_001083308 | rs377044348 | c.245C>T (p.Thr82Met) | 0.0000178 | - | P | - | B | - |  |  |  | LB |
| VITT18 | TXNIP NM_006472 | rs34791738 | c.520C>T (p.Pro174Ser) | 0.0085 | - | - | - | - | - |  |  |  | LB |
| VITT18 | GSDMD NM_024736 | rs138749323 | c.746G>A (p.Arg249His) | 0.000301 | T | P | N | D | T |  |  |  | B |
| VITT18 | POMT2 NM_013382 | rs138488805 | c.2148-18A>G | 0.00608 | - | - | - | - | - | NEG | NEG | NEG | B |
| VITT18 | AIMP NM_00114241 | rs138106524 | c.592C>T (p.Pro198Ser) | 0.000607 | T | D.C. | D | B | D |  |  |  | LB |
| VITT18 | FPR2 NM_001005738 | rs74602258 | c.220T>C (p.Phe74Leu) | 0.00662 | T | D.C. | D | P.D. | D |  |  |  | LB |
| VITT18 | NLRP9 NM_176820 | rs80009430 | c.126C>G (p.Ile42Met) | 0.00379 | T | P | N | D | D |  |  |  | LB |
| **VITT021** | **LRTM2 NM_001163926** | **rs919752328** | **c.1046G>A (p.Arg349His)** | **N/A** | **T** | **D.C.** | **N** | **D** | **D** |  |  |  | **VUS** |
| **VITT021** | **COL5A1 NM_001278074** | **rs201997623** | **c.404C>T (p.Ser135Phe)** | **0.00001763** | **T** | **D.C.** | **D** | **D** | **D** |  |  |  | **VUS** |
| **VITT21** | **ADAM8 NM_001109.5** | **rs368130036** | **c.923C>T (p.Ala308Val)** | **0.000121** | **T** | **P** | **D,N,D** | **P.D.** | **D** |  |  |  | **VUS** |
| **VITT21** | **JMJD7-PLA2G4B NM_005090.4** | **rs146887953** | **c.66G>T (p.Glu22Asp)** | **0.00103** | **T** | **P** | **N** | **B** | **T** |  |  |  | **VUS** |
| VITT21 | SERPINA10 NM_016186 | rs2232708 | c.811G>A (p.Gly271Ser) | 0.006813 | D | P | N | B | T |  |  |  | LB |
| VITT21 | BRCA2  NM_000059 | rs80359143 | c.8918G>A (p.Arg2973His) | 0.00003556 | T | P | N | P.D. | D |  |  |  | LB |
| VITT21 | ADRA2C  NM_000683 | rs1346646919 | c.942_950del (p.Gly316_Ala318del) | 0.004276 | - | - | - | - | - |  |  |  | B |
| VITT21 | SERPINA10  NM_016186.2 | rs2232698 | c.262C>T (p.Arg88Ter) | 0.006544 | - | - | - | - | - |  |  |  | B |
| VITT21 | ZNF304  NM_020657.4 | rs45619942 | c.280T>C (p.Cys94Arg) | 0.007966 | T | P | D | B | D |  |  |  | B |
| VITT21 | LAMA5  NM_005560 | rs111653839 | c.7114G>A (p.Asp2372Asn) | 0.009467 | T | P | N | B | T |  |  |  | B |
| VITT21 | CD2AP NM_012120.3 | rs750459384 | c.1727A>G (p.Asp576Gly) | 0.0000617 | T | D.C. | N | B | T |  |  |  | LB |
| VITT21 | CHST4 NM_001166395.2 | rs117029243 | c.1078C>T (p.Arg360Cys) | 0.00575 | D | D.C. | D | D | D |  |  |  | LB |
| VITT21 | JMJD7-PLA2G4B NM_005090.4 | rs141583854 | c.2809C>T (p.Arg937Trp) | 0.000838 | T | P | D | D | D |  |  |  | LB |
| VITT21 | PLAA NM_001031689.3 | rs140970730 | c.1445C>T (p.Ser482Leu) | 0.00112 | T | D.C. | D | B | D |  |  |  | LB |
| VITT21 | FCER1A NM_002001.4 | rs2298805 | c.302G>A (p.Ser101Asn) | 0.000918 | - | - | - | - | - |  |  |  | B |
| VITT21 | HLA-DRB1 NM_002124.4 | rs35445101 | c.790T>C (p.Phe264Leu) | 0.000537 | T | P | N | B | T |  |  |  | B |
| VITT21 | SELP NM_003005.4 | rs115596475 | c.1334-3T>C | 0.000205 | - | - | - | - | - | NEG | NEG | NEG | B |
| VITT21 | LACC1 NM_001128303.2 | rs34414396 | c.112A>G (p.Lys38Glu) | 0.00585 | T | P | N | B | T |  |  |  | LB |
| VITT21 | SELE NM_000450.2 | rs3917422 | c.770A>C (p.Gln257Pro) | 0.00022 | T | P | N | B | T |  |  |  | LB |
| VITT21 | TLR3 NM_003265.3 | rs35311343 | c.889C>G (p.Leu297Val) | 0.00214 | T | D.C. | N | D | D |  |  |  | LB |
| VITT21 | MFHAS1 NM_004225.3 | N/A | c.84C>A (p.Asn28Lys) | N/A | T | D.C. | N | B | D |  |  |  | LB |
| VITT21 | SCYL3 NM_181093.4 | rs55788990 | c.2059G>A (p.Ala687Thr) | 0.00081 | T | P | N | P.D. | D |  |  |  | LB |
| VITT21 | CD5L NM_005894.3 | rs1557941669 | c.56-11T>C | 0.00000419 (gMAF) | - | - | - | - | - | NEG | NEG | NEG | LB |
| VITT21 | PXK NM_017771.5 | rs145159214 | c.1103-19T>C | 0.00338 | - | - | - | - | - | NEG | NEG | NEG | LB |

**FATHMM**: T=Tolerated, D=Damaging

**Mutation Taster**: P=Polymorphism; D.C.=Disease Causing

**Provean**: N=Neutral; D=Damaging

**Polyphen**: B=Benign Variant; P.D.=Possibly/Probably Damaging; D=Damaging

**SIFT**: T=Tolerated; D=Damaging

**ACMG classification**: **VUS**= Variant of Uncertain Clinical Significance; **LB**= Likely Benign Variant, **B**=Benign Variant
